# Sonoporation-induced cell membrane permeabilization and cytoskeleton disassembly at varied acoustic and microbubble-cell parameters

**DOI:** 10.1038/s41598-018-22056-8

**Published:** 2018-03-01

**Authors:** Maochen Wang, Yi Zhang, Chenliang Cai, Juan Tu, Xiasheng Guo, Dong Zhang

**Affiliations:** 1 Key Laboratory of Modern Acoustics (MOE), Department of Physics, Collaborative Innovation Centre of Advanced Microstructure, Nanjing University, Nanjing, 210093 China; 2 The State Key Laboratory of Acoustics, Chinese Academy of Science, Beijing, 10080 China

## Abstract

Sonoporation mediated by microbubbles has being extensively studied as a promising technique to facilitate gene/drug delivery to cells. Previous studies mainly explored the membrane-level responses to sonoporation. To provide in-depth understanding on this process, various sonoporation-induced cellular responses (e.g., membrane permeabilization and cytoskeleton disassembly) generated at different impact parameters (e.g., acoustic driving pressure and microbubble-cell distances) were systemically investigated in the present work. HeLa cells, whose α-tubulin cytoskeleton was labeled by incorporation of a green fluorescence protein (GFP)-α-tubulin fusion protein, were exposed to a single ultrasound pulse (1 MHz, 20 cycles) in the presence of microbubbles. Intracellular transport via sonoporation was assessed in real time using propidium iodide and the disassembly of α-tubulin cytoskeleton was observed by fluorescence microscope. Meanwhile, the dynamics of an interacting bubble-cell pair was theoretically simulated by boundary element method. Both the experimental observations and numerical simulations showed that, by increasing the acoustic pressure or reducing the bubble-cell distance, intensified deformation could be induced in the cellular membrane, which could result in enhanced intracellular delivery and cytoskeleton disassembly. The current results suggest that more tailored therapeutic strategies could be designed for ultrasound gene/drug delivery by adopting optimal bubble-cell distances and/or better controlling incident acoustic energy.

## Introduction

Sonoporation, a microbubble-medicated biophysical process, has shown great potential to facilitate the delivery of drugs, genes and other therapeutic agents into cell^1–6^, by transiently perforating the plasma membrane to enhance the membrane permeability^7–10^. Heterogeneous cellular responses have also been observed, such as calcium-ion transients^11,12^, depolarization of plasma membrane potential^13^, temporary neurite retraction and cell body shrinkage^14^. Moreover, recent studies have demonstrated that sonoporation could disrupt actin cytoskeleton organization^15,16^ and induce cell nucleus contraction^17^, which indicate that sonoporation is a holistic and complex change, instead of a sole membrane-level phenomenon.

Microbubbles play a crucial role in the process of sonoporation, as the formation of high-speed jet or the cavitation-induced localized displacement of cellular membrane is one of the major mechanisms of sonoporaiton^18–21^. It has been demonstrated that the sonoporation outcomes could be significantly affected by both acoustic driving parameters and microbubble-to-cell relative parameters^22,23^. For instance, it has been demonstrated that the sonoporation pore size is highly correlated with acoustic driving parameters, *e.g*., acoustic pressure, sonication duration time and pulse repetition frequency^11,24–26^. Heterogeneous cellular membrane permeability variations have also been observed at different microbubble-cell distance^27,28^, bubble size^23^ and microbubble-cell numbers^29,30^. However, these studies mainly explored the membrane-level responses to microbubble-mediated sonoporation and did not fully illuminate the impacts of acoustic driving parameters and microbubble-to-cell relative parameters on deeper cellular structures, such as the cytoskeleton.

Microtubule cytoskeleton, polymerized by α-tubulins^31^, play an important role in many cellular processes, including structural support, mitosis and intracellular transport^32,33^. Fan *et al*. observed the impact of cell cycle phase on the membrane permeabilization and microtubule cytoskeleton disassembly induced by sonoporation^34^. Zeghimi *et al*. also reported that sonoporation induces the rearrangement of actin and tubulin cytoskeleton and this reorganization is transient^35^. So far, these studies on the involvement of cytoskeleton were all carried with fixed acoustic driving parameters and microbubble-to-cell relative parameters. Therefore, more efforts deserve to be made to simultaneously examine sonoporation-induced cellular responses in both cell membrane and cytoskeleton, at various acoustic driving conditions and microbubble-cell relative parameters.

The main goal of the current study was to systematically investigate the mechanisms relative to how the cellular responses were affected by acoustic and microbubble-cell parameters. Here, *in-situ* cellular responses to microbubble-mediated sonoporation process generated with different parameters were systemically visualized based on an integrated system combining ultrasound exposure apparatus with real-time fluorescence microscope imaging. Based on the real-time experimental observation, the impacts of acoustic driving pressure and microbubble-cell distance on cellular responses, such as the α-tubulin cytoskeleton disassembly and the membrane permeabilization, were quantitatively analyzed. Although the exact mechanism involved in the sonoporation process has not been fully understood due to the complexity of ultrasound-mediated interactions between cell and microbubbles, it has been hypothesized that the jet excited by the bubble collapse may play an important role in the sonoporation process^18,22,36–38^. Therefore, a two-dimensional (2D) boundary element method (BEM) model was developed to simulate microbubble-cell interaction^19,39,40^ and further discussions were made by comparing the current experimental observations with previous theoretical simulation results.

The current results would be beneficial for getting in-depth understanding of the mechanism involved in the process of microbubble-mediated sonoporation, which could enable more tailored therapeutic strategies for ultrasound gene/drug delivery facilitated by microbubble-mediated sonoporation.

## Results

### Cellular responses induced by microbubble-mediated sonoporation

**Figure 1:**
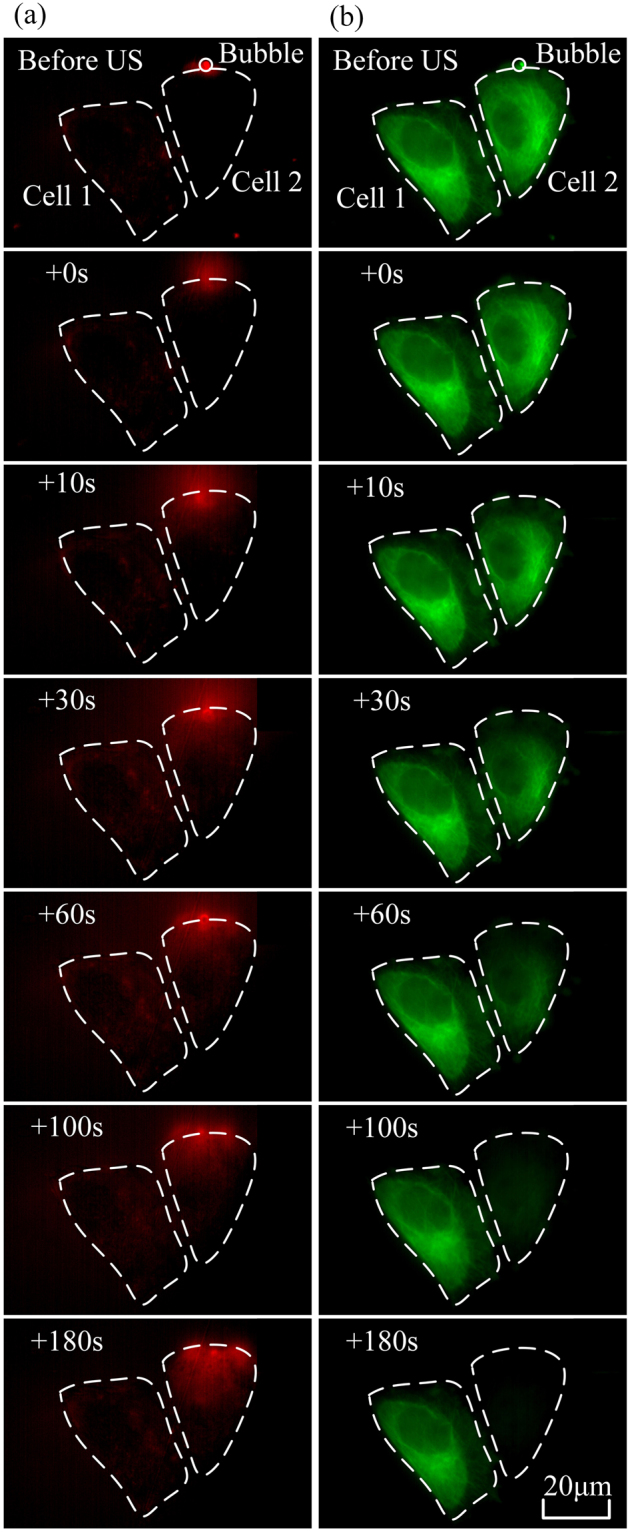
Intracellular uptake of PI and cytoskeleton disassembly are spatially correlated with stimulation of microbubbles-mediated sonoporation. (**a**) Time-lapse PI fluorescence images and (**b**) α-tubulin cytoskeleton images. The dashed lines show the regions of interest (Cell 1 and Cell 2), while the circle outlines the microbubble next to Cell 2 at the beginning of the experiment.

In the present work, human cervical epithelial carcinoma (HeLa) cells, whose α-tubulin cytoskeleton was labeled by incorporation of a green fluorescence protein (GFP)-α-tubulin fusion protein (referred as GFP-α-tubulin HeLa cells) were used in the experiments. Figure 1 shows a time-series rendering of this observation based on live images acquired using our platform. The green fluorescence depicts the GFP-α-tubulin cytoskeleton networks and the red fluorescence indicates intracellular uptake of propidium iodide (PI) that serves as the sonoporation tracer. The boundaries of two cells are labeled as dash lines and the position of the pre-exposure microbubble is indicated by a white circle. Only those microbubbles adjacent to cells (*e.g*., Cell 2) could result in any observable changes in cellular structures. We can see that the PI delivery originates from the immediate vicinity of the microbubble at the onset time of the ultrasound exposure and then diffuses to the other areas of Cell 2 over the next 180-s, while the cell without bubbles nearby (*e.g*., Cell 1) is entirely unaffected. It was also noticed in the experiments that the disassembly of the cytoskeleton reflected by the decay of green fluorescence would simultaneously occur with the rupture of cell membrane upon the onset of microbubble-mediated sonoporation, which indicates that sonoporated cells underwent a variety of complex changes in their cellular structures.

### Fluorescence observations on cellular responses induced by sonoporation at different acoustic and microbubble-cell parameters

**Figure 2:**
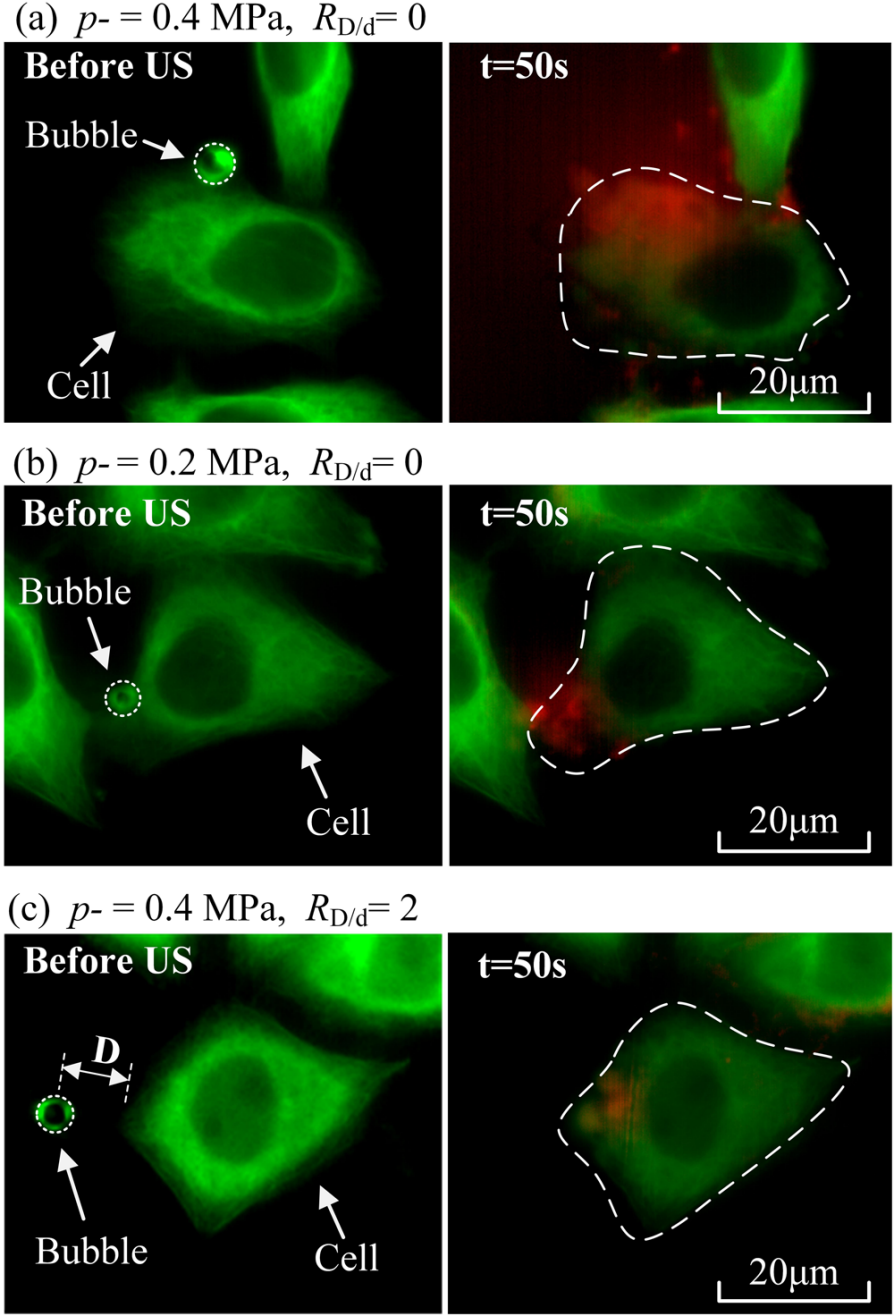
Fluorescent images before and after sonoporation (*t = *0 and 50 s, respectively) with different acoustic pressures and microbubble-cell parameters. (**a**) Microbubble-cell interaction happening at *p*− = 0.4 MPa and *R*_D/d_ = 0; (**b**) Microbubble-cell interaction happening at *p*− = 0.2 MPa and *R*_D/d_ = 0; and (**c**) One microbubble locating near a cell (*R*_D/d_ = 2) and driven at *p*− = 0.4 MPa.

The main aim of the current investigation was to analyze the disassembly of the cytoskeleton and the enhancement of membrane permeability in circumstances of acoustic and microbubble-cell parameters. The microbubble-cell distance (*D*) was set to be the minimum distance between the center of a microbubble and the cell membrane and the microbubble-cell parameter (*R*_D/d_) was determined by the ratio between the microbubble-cell distances (*D*) and the diameters (*d*) of microbubbles, viz., ${R}_{\text{D}/\text{d}}=\frac{D}{d}$. For simplifying the analysis, we calculated microbubble-cell parameters and divided the data into 3 groups that the *R*_D/d_ ratios are 0, 1 and 2, by rounding up the ratios. The experiments were divided into two categories: (1) the peak negative acoustic driving pressure (*p*- varied at 0 (viz., sham), 0.2, 0.3 and 0.4 MPa, with fixed *R*_D/d_ = 0; and (2) the microbubble-cell parameter *R*_D/d_ varied at 0, 1 and 2, with fixed peak negative pressure *p*− = 0.4 MPa. Some sample fluorescent images of cellular response induced by microbubble-mediated sonoporation at different acoustic pressures and microbubble-cell distances are shown in Fig. 2. The comparison between Fig. 2a and b suggests that more intensified cytoskeleton disassembly and PI uptakes are induced at relatively higher acoustic driving pressure. By comparing Fig. 2a and c, it is also observed that sonoporation-induced cellular responses could be impaired when the bubble gets closer to the cell.

### Quantitatively analyses of cellular responses induced by sonoporation at different acoustic and microbubble-cell parameters

To quantitatively analyze the sonoporation process, the α-tubulin cytoskeleton and PI fluorescence intensity curves were fitted respectively, following a previously proposed model^12,41,42^. The green fluorescence intensity decay due to the disassembly of the cytoskeleton was fitted to a two-parameter exponential decay function

1 $$I(t)={I}_{\infty }+({I}_{0}-{I}_{\infty })\exp (-\,\frac{t}{{\tau }_{\text{GFP}}})$$

where *τ*_GFP_ was the characteristic decay time for cytoskeleton disassembly; *I*_0_ and *I*_∞_ were the initial cytoskeleton intensity at 476 nm excitation before ultrasound exposure and the asymptotic cytoskeleton intensity after a long time, respectively. Analogously, the red fluorescence intensity enhancement due to enhanced PI uptake was fitted to a two-parameter exponential recovery function

2 $$PI(t)=P{I}_{\infty }+(P{I}_{0}-P{I}_{\infty })\exp (-\,\frac{t}{{\tau }_{\mathrm{PI}}})$$

where *τ*_PI_ is characteristic PI recovery time; *PI*_0_ and *PI*_∞_ are the initial PI intensity and the asymptotic PI intensity respectively. The fitting process was performed using the Origin (OriginLab Co. Northampton, MA, USA) Curve Fitting Tool.

**Figure 3:**
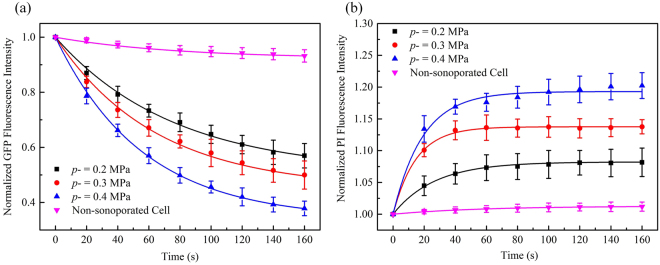
Temporal evolution curves of (**a**) GFP and (**b**) PI fluorescence fitted with exponential decay and recovery functions, with different peak negative pressures *p*− and fixed *R*_D/d_ = 0. The number of sonoporated cells observed under individual pressures is *n* = 15. Meanwhile, a total of 30 non-sonoporated cells close to the sonoporated ones are also analyzed.

The dependence of cellular responses induced by sonoporation on acoustic driving pressure was examined in this experiment at *p*− = 0.2, 0.3 and 0.4 MPa and the fluorescence intensity of non-sonoporated cells was also recorded for comparison (viz. *p*− = 0 MPa). Other acoustic driving parameters remained the same and the microbubble-cell parameter was kept to be *R*_D/d_ = 0. The results of normalized GFP fluorescence intensity shown in Fig. 3a indicate that, by increasing the amplitude of acoustic pressure, the GFP-α-tubulin cytoskeleton would undergo more rapid disassembly. At the moment of 160-s, when the acoustic pressure reached 0.4 MPa, the GFP fluorescence intensity was less than two thirds of the intensity at the pressure of 0.2 MPa. In contrast, the GFP fluorescence intensity of cells that didn’t experience the sonoporation process attenuated quite slowly.

Figure 3b shows the sonoporation-triggered temporal curves of PI fluorescence intensity. HeLa cells exhibited significantly slower PI growth rate when the acoustic pressure was reduced, which suggested that, for single-site microbubble sonoporation, these cells underwent the least enhanced membrane permeabilization. Similar to the cases of GFP intensity, the PI intensity of non-sonoporated cells showed that almost no PI entered these cells.

**Figure 4:**
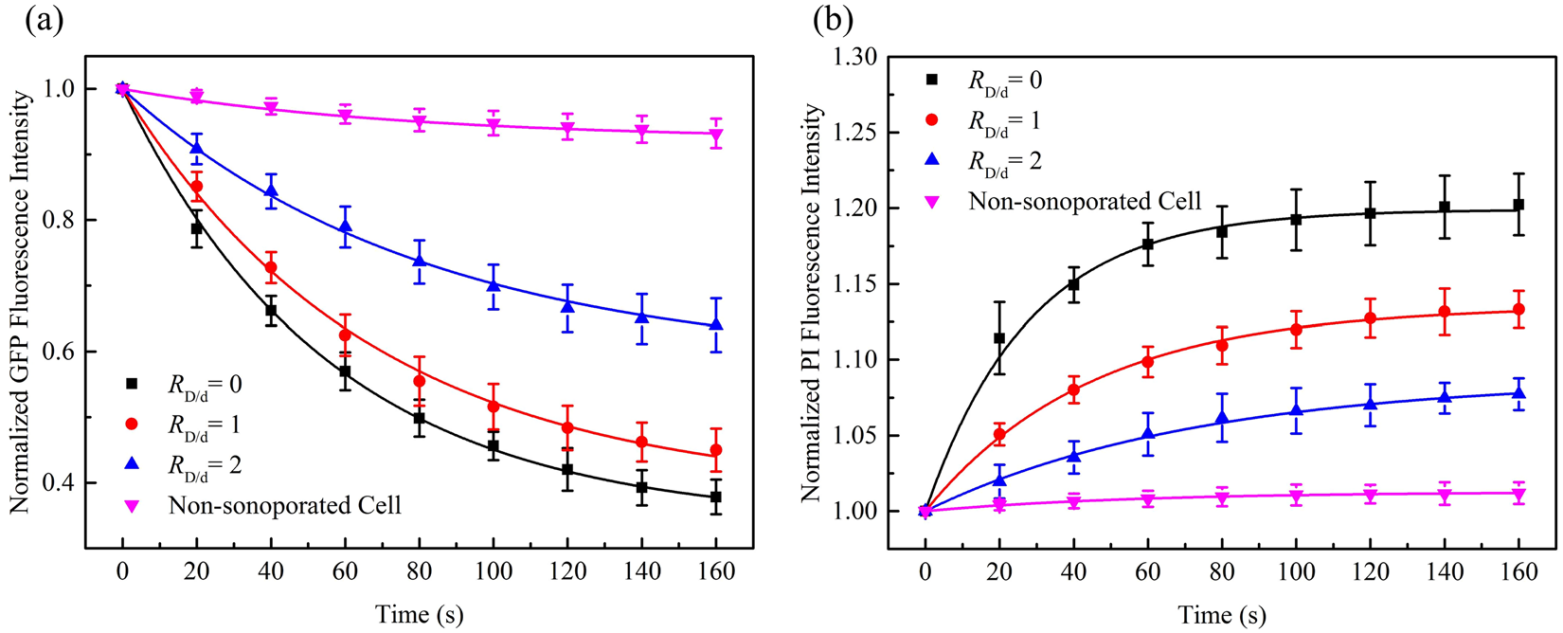
Temporal evolution curves of (**a**) GFP and (**b**) PI fluorescence fitted with exponential decay and recovery functions, with different microbubble-cell parameters *R*_D/d_ and constant *p*− = 0.4 MPa. The number of sonoporated cells observed with individual *R*_D/d_ is *n* = 15. Meanwhile, a total of 30 non-sonoporated cells close to the sonoporated ones are also analyzed.

In this series of experiments, sonoporation-induced cellular responses were observed at various microbubble-cell parameters, while the peak negative acoustic driving pressure was kept at 0.4 MPa. In other words, the bubbles and cells might not attach to each other, which resulted in different bubble-cell spatial distances. Figure 4a shows the temporal traces of normalized cytoskeleton fluorescence for the cells in the cases of three different microbubble-cell distance ratios (viz., *R*_D/d_ = 0, 1, 2) over a 160-s period. As can be observed, cells all exhibited cytoskeleton disassembly with different microbubble-cell distances, but they exhibited diversity in their cytoskeleton disassembly level over time. When the *R*_D/d_ was smaller, namely the microbubble located closer to the cell membrane at the instant of bubble rupture, the α-tubulin cytoskeleton would undergo more rapid and intense disassembly. Meanwhile, it was also observed that, for all cells regardless of microbubble-cell distances, the disassembly rate of α-tubulin cytoskeleton would decelerate from the moment of perforation.

Figure 4b shows time traces of normalized PI fluorescence for the cells in the cases of three different microbubble-cell distance ratios (*R*_D/d_ = 0, 1, 2) over a 160-s period. All sonoporated cells exhibited PI uptake at different bubble-cell distances, but they exhibited differences in their PI uptake level over time. At smaller *R*_D/d_, more PI was uptaken into the cells at faster influx rate.

## Discussions

The GFP-α-tubulin HeLa cells used in the present work can stably express green fluorescence with an intact microtubule network^34^. As part of the cytoskeleton network, microtubules are long, hollow cylinders composed up of polymerized α- and β-tubulin dimers, which can be found throughout the cytoplasm in the cell^43,44^. Sonoporation may disrupt the integrity of the microtubule cytoskeleton and induce irreversible disassembly in α-tubulin network^34^. Chen *et al*. also reported that single-site sonoporation could lead to the rupture of F-actin network adjacent to the perforation site and enhanced disintegration of F-actin into its globular monomer form was observed^15^. Consistent with previous reports, simultaneous cytoskeleton disassembly was clearly observed with the permeabilization of the cell membrane in the current work.

Based on the model mentioned above, the temporal evolutions of α-tubulin cytoskeleton arrangement and PI uptake could be well fitted. By analyzing the fitted parameters, the concurrent activity between GFP-α-tubulin cytoskeleton disassembly and PI uptake through the perforated membrane could be noticed.

**Figure 5:**
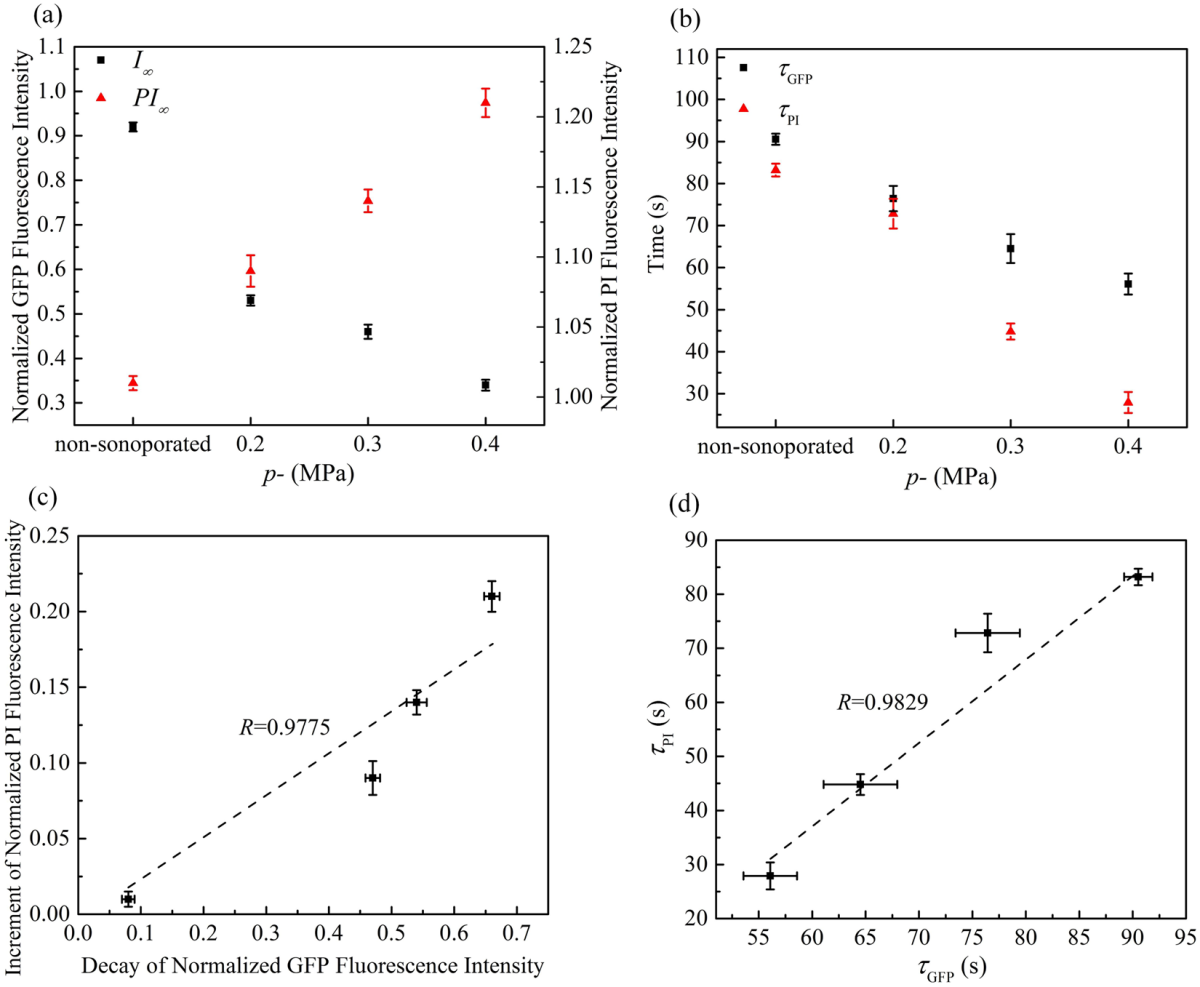
Quantitatively analyses of α-tubulin cytoskeleton arrangement and PI uptake induced by sonoporation with different peak negative pressures *p*− and fixed *R*_D/d_ = 0: (**a**) The values of asymptotic fluorescence intensity; (**b**) the values of characteristic decay time and recovery time; (**c**) a correlation plot showing the relationship between increment of PI fluorescence intensity and decay of GFP fluorescence intensity; and (**d**) a correlation plot showing the relationship between *τ*_GFP_ and *τ*_PI_. Linear correlation coefficients are also indicated.

As shown in Fig. 5(a), one can see that cells undergoing sonoporation at higher acoustic pressure will have a smaller asymptotic GFP intensity (*I*_∞_), which suggests the α-tubulin cytoskeleton network should undergo relatively severe disruption when we increased the acoustic energy. The characteristic decay time (*τ*_GFP_) represents the time that the α-tubulin cytoskeleton of individual cells will assume to disassemble until reaching a stable plateau. Therefore, as shown in Fig. 5(b), smaller acoustic pressure would result in longer decay time for the disassembly of the cell cytoskeleton. Meanwhile, larger asymptotic intensity (*PI*_∞_) and shorter characteristic PI recovery time (*τ*_PI_) can also be observed at higher acoustic pressure, which indicates that the permeability of the cell membrane should be enhanced significantly so that more PI could influx into the cell. The increment of normalized PI fluorescence intensity and the decay of normalized GFP fluorescence intensity were calculated quantitatively, viz., |I_∞_ − I_0_| and |*PI*_∞_ − *PI*_0_|, respectively. As shown in Fig. 5(c), the increment of PI and the decay of GFP are positively correlated (linear correlation coefficient *R* = 0.9775), indicating that sonoporation could increase the level of α-tubulin cytoskeleton disassembly and membrane permeabilization simultaneously. Moreover, the plot in Fig. 5(d) shows a positive correlation between the characteristic decay time for cytoskeleton disassembly and characteristic PI recovery time (linear correlation coefficient *R* = 0.9829).

**Figure 6:**
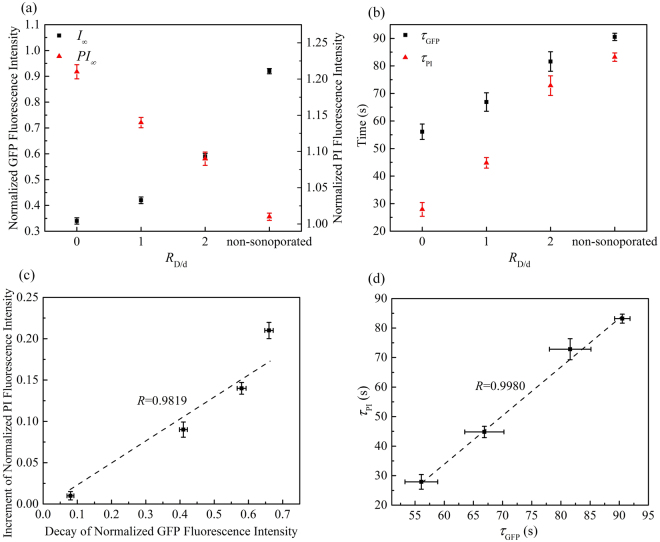
Quantitatively analyses of α-tubulin cytoskeleton arrangement and PI uptake induced by sonoporation with different microbubble-cell parameters *R*_D/d_ and constant *p*− = 0.4 MPa: (**a**) The values of asymptotic fluorescence intensity; (**b**) the values of characteristic decay time and recovery time; (**c**) a correlation plot showing the relationship between increment of PI fluorescence intensity and decay of GFP fluorescence intensity; and (**d**) a correlation plot showing the relationship between *τ*_PI_ and *τ*_GFP_ . Linear correlation coefficients are also indicated.

Similarly, as shown in Fig. 6, relatively severe disruption of α-tubulin cytoskeleton network and significantly enhanced membrane permeabilization could be observed, when the bubble gets closer to the cell. Figure 6(c) and (d) show that, for the variations of fluorescence intensity and the characteristic time of GFP decay and PI growth, positive correlations can be observed (viz., *R* = 0.9819 and R = 0.9980, respectively).

These phenomena suggest that, for rapid intracellular delivery facilitated by microbubble-mediated sonoporation, the enhancement rate of membrane permeability usually correlates with the disassembly rate of cytoskeleton network. The α-tubulin microtubules are typically located in the deep portions of the cell cytoplasm^34^, so the mechanical effect induced by sonoporation could be transmitted into the deeper cellular structures to trigger more complicated bioeffects on cells.

It has been reported that sonoporation is in essence an act of traumatizing the plasma membrane on an acute but transitory basis^45^ and is not solely a membrane-level phenomenon^15^. Although the exact mechanism underlying the sonoporation process has not been fully understood due to complicated dynamics involved in microbubble-cell interactions, the violent collapse of inertial cavitation bubble near the cell membrane is regarded as one of most important mechanisms underlying the process of sonoporation^46–51^. To provide comprehensive explanation for the phenomenon observed in the experiments, boundary element method (BEM) simulations were carried out to simulate the dynamic response of an interacting bubble-cell pair^39^.

BEM modelling was performed using a commercial 3D fluid flows simulation code package 3DynalFS-BEM (DynaFlow Inc., Jessup, MD, USA). It was assumed that an interacting bubble-cell pair was surrounded by infinite liquid medium. Before the ultrasound was triggered, the bubble and the cell were spherical at their equilibrium states. In the modeling, both the bubble and the cell were discretized based on a spherically symmetric scheme. In order to simulate the coupling between the sphere structures and the surrounding fluid, locations of all surface nodes can be achieved with a structural module in the code package^39^.

**Figure 7:**
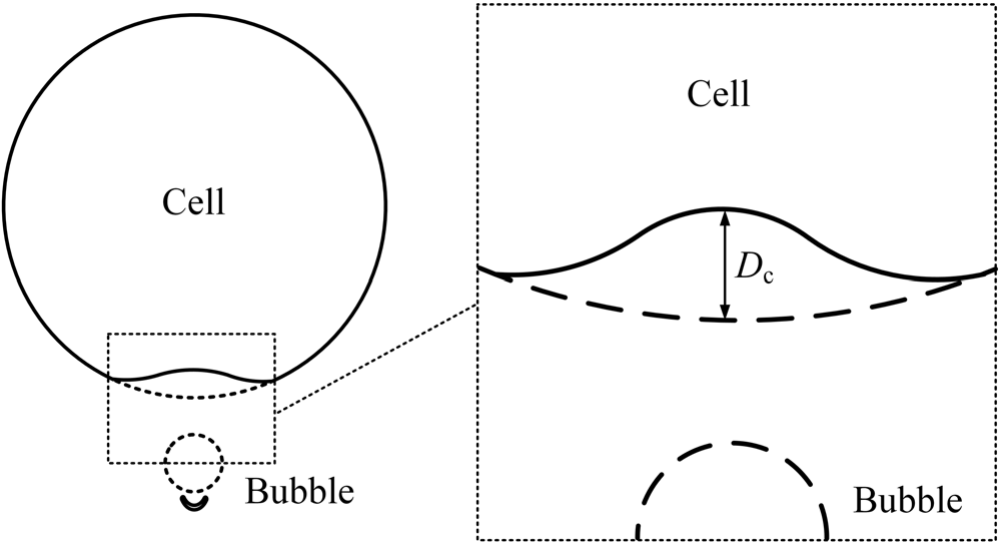
Illustration of the bubble-cell interaction.

Previous experimental observations have demonstrated that the oscillation of sonicated microbubbles might cause cell deformation by “pushing/pulling” cell membranes^19–21,52^. To further understand the interactions between ultrasound-driven microbubbles and cells, the morphological evolvement around the close-to-bubble point (CP) on cell membrane was monitored^52^. As shown in Fig. 7, the dashed line depicts the outlines of the bubble and cell at their equilibrium states and the solid line indicates the cell’s deformations before the rupture of the bubble. The right part of the figure is an enlarged view of the area around CP, in which *D*_c_ was defined as the displacement of CP.

**Figure 8:**
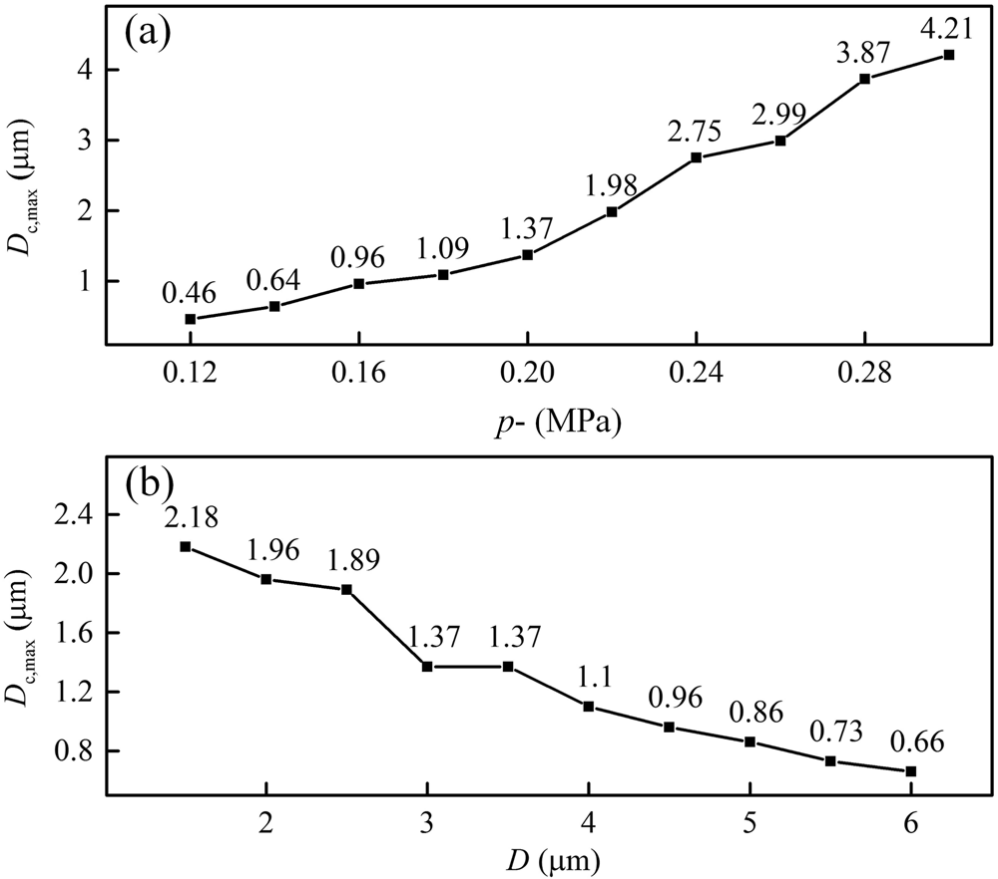
(**a**) Influence of acoustic pressure on the maximum CP displacement, (**b**) Influence of initial bubble-cell distance on the maximum CP displacement (*f* = 1 MHz).

Parametric studies were carried out, revealing the impacts of acoustic pressure and microbubble-cell distance parameter on cellular deformation. As shown in Fig. 8a, by fixing the ultrasonic frequency to 1 MHz and the bubble-cell distance to 3 μm, at an acoustic pressure of 0.12 MPa, the maximum CP displacement was less than 0.5 μm. However, if the acoustic pressure is raised to 0.3 MPa, much more severe deformation could be generated in the cell membrane, which would give a *D*_c_ of 4.21 μm, overtaking the initial bubble-cell distance. The results were consistent with the prevalent viewpoints that the bubble-cell interaction got stronger with increasing acoustic pressure^20,22,53^ and agreed well with our experimental results.

Apart from the acoustic pressure, the distance between the bubble and cell might also have significant impact on collapse-induced deformation in cellular structures. By fixing the ultrasonic frequency to 1 MHz and the peak negative acoustic pressure to 0.2 MPa, the bubble-cell distance was set to vary from 1.7 μm to 6.0 μm. Figure 8b shows that, as the bubble and cell were separated further from each other, the cell membrane deformation, reflected by *D*_c_, gradually decreased from 2.18 μm to 0.66 μm. The interaction between the bubble and the cell weakened due to the widening gap, increasing the possibility of failing to penetrate the cell. This result coincided well with experimental observations.

It was reported that the pulse length might also have a major impact on cellular dynamics during the process of sonoporation^22,46,54,55^. A single shock-wave exposure could lead to the cellular molecular uptake and the detachment of cells^46^. Kudo *et al*. reported that a single-shot pulsed ultrasound of 3 cycles could perforate endothelial cell membrane, when bubbles of resonant sizes were adjacent to cells^56^. If the acoustic pressure kept constant, an increase in the pulse duration should cause a decrease in the cell viability^57–59^. For instance, Fan *et al*. evaluated the uptake of fluorescently labeled pDNA in the ultrasound treated cells at two different pulse lengths: 10 cycles and 10^5^ cycles, both with an acoustic pressure of 400 kPa^59^. They claimed that the short ultrasonic pulses resulted in relatively high delivery rates (30%) and higher cell viabilities (50%), while the long pulses led to low delivery rates (10%), large cell membrane disruptions and massive cell death (90%)^59^. Our present work focused on the impacts of acoustic driving pressure and microbubble-cell distance on cellular responses and further efforts will be made to investigate the effect of pulse duration on the sonoporation outcome in future experiments.

The goal of this study is to investigate that how the acoustic pressure and bubble-cell distances influence the cell dynamics, such as the α-tubulin cytoskeleton and the cellular membrane permeability. We measured the spatiotemporal PI intensity and cytoskeleton intensity of living cells together in real time and quantitatively measured the recovery time constants and fluorescence intensities. The role of ultrasound-induced microbubble changes was verified by the acquisition of images of the same bubble and cells, before, during and immediately after a short ultrasound pulse. The results show that as increasing the pressure amplitude, or the microbubbles get closer to cells, the disassembly of the cytoskeleton and the increase of cellular membrane permeability will accelerate.

## Conclusions

Here, based on an integrated system combining ultrasound exposure apparatus with real-time fluorescence microscopic imaging, *in-situ* cellular responses (viz., cytoskeleton disassembly and intracellular delivery) induced by microbubble-mediated sonoporation were assessed at varied acoustic pressures and microbubble-cell distances. In addition, a two-dimensional (2D) boundary element method (BEM) model was developed to simulate microbubble-cell interactions, especially the morphological characteristics around the close-to-bubble point (CP) on cell membrane. The results show that the deformation of CP on the cell membrane could be intensified with raised acoustic pressure or reduced bubble-cell distance, so that the cell membrane and cytoskeleton would undergo greater damage. The results suggest that, in order to boost more efficient and safer sonoporation-related treatments, it is better to find optimal bubble-cell distance and appropriately control acoustic pressure according to different therapeutic circumstances.

## Materials and Methods

### Cell preparation

HeLa cervical cancer cells (provided by the medical school at Nanjing University) labeled by a GFP-α-tubulin fusion protein^60^ (excitation/emission maxima: 476/510 nm) were chosen as the cell line model for the experiments, because it is easy to perform real-time visualization on the variation of microtubule cytoskeleton arrangement. In this work, HeLa cells were first grown inside a Petri dish in an incubator environment (37 °C with 5% carbon dioxide). The culturing medium was based on Gibco’s RPMI medium 1640 (Carlsbad, CA, USA) with 2.5% fetal bovine serum supplements (Sigma-Aldrich, St. Louis, MO, USA). Before the experiment, cells were harvested by Trypsin–EDTA (Gibco Inc., Carlsbad, CA, USA) and transferred to an OptiCell™ chamber (Nunc, Rochester, NY, USA) filled with the same cell culture medium. OptiCell™ chambers consist of two parallel gas-permeable cell-culture-treated, polystyrene membranes (50 cm^2^, 75 μm thick, 2 mm apart) attached to a standard microtiter plate-sized frame.

### Experimental system

**Figure 9:**
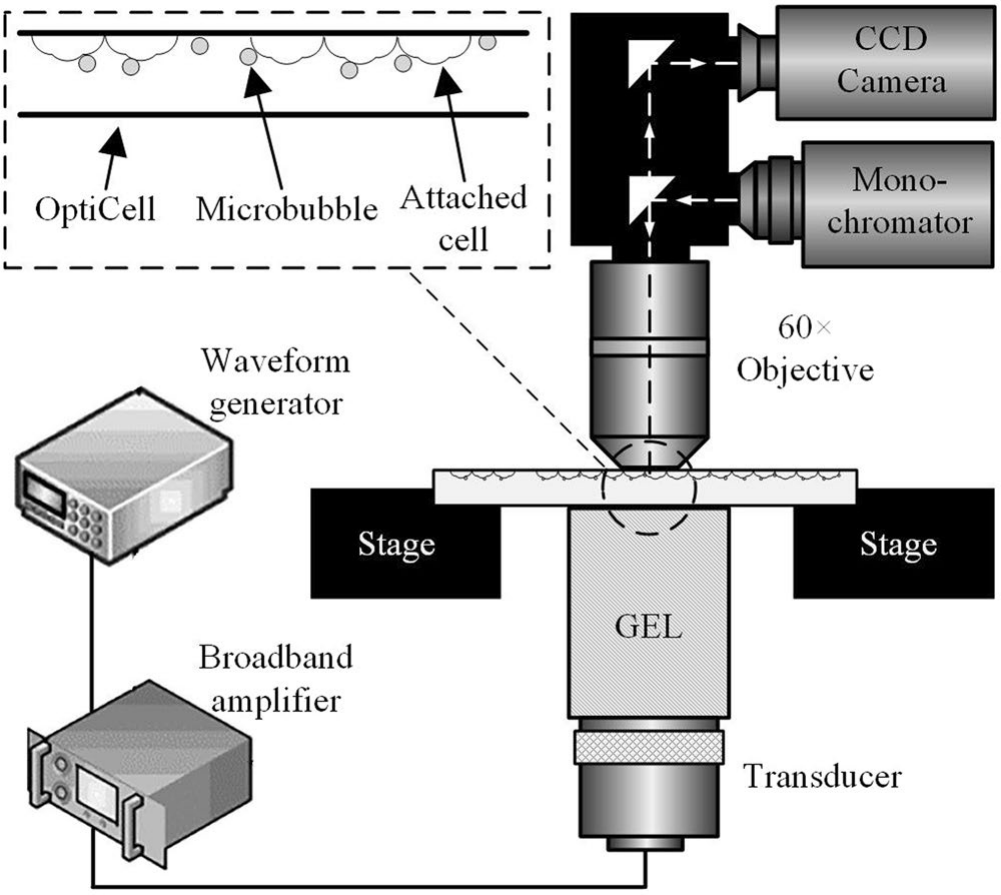
A schematic of the acoustic exposure apparatus used to investigate intracellular delivery of fluorescent marker and cytoskeleton dynamics induced by sonoporation.

The disassembly of cytoskeleton and the variation of cell membrane permeability were investigated at different acoustic driving pressures and microbubble-cell distances, by performing live cell imaging during the process of sonoporation. As shown in Fig. 9, the ultrasound exposure apparatus is comprised of a cascade of an arbitrary waveform generator (33250 A, Agilent, Palo Alto, CA, USA), a broadband amplifier (2200 L, Electronics Innovation, Rochester, NY, USA) and a single-element focused piston transducer (1 MHz center frequency, A314S, Olympus Panametrics-NDT, Waltham, MA, USA). Ultrasound pulses emitted from the ultrasound exposure apparatus passed through a customized cylindrical polyacrylamide gel to focus on the HeLa cells, which grew on the upper wall of an OptiCell™ chamber. During the experimentation, the cell morphology was observed in real time using a fluorescence microscope (BX53, Olympus, Shinjuku, Tokyo, Japan) with a 60× oil immersion lens.

In this study, the modulating frequency of the arbitrary waveform generator was set to 1 MHz, which is the same as the transducer’s center frequency. The applied pulse was a 20-cycle tone burst (i.e., 20 μs pulse duration) and the pulse triggering was manually performed to facilitate single-pulse ultrasound activation. The *in-situ* acoustic peak negative pressure at the focus was calibrated to be 0.2 MPa, 0.3 MPa and 0.4 MPa, by using the NTR needle hydrophone (TNU001A, NTR Systems Inc., Seattle, WA, USA).

SonoVue microbubbles (Bracco diagnostics Inc., Geneva, Switzerland) with an average diameter of ~1.5 µm were used as sonoporation agents in this work. After resuspension of the microbubbles in 5-ml phosphate buffer solution according to the manufacturer’s instruction, the bubbles were added to the OptiCell™ sample holder in diluted form at an approximate concentration of 6 × 10^6^ bubbles/ml. To ensure that the microbubbles were distributed close to cells at the start of ultrasound exposure, the OptiCell™ sample holder was flipped upside down so that the cell monolayer would be at the top side and the microbubbles would rise to the top by the buoyancy forces. The microbubbles randomly dispersed in the cell culture medium and the distances between microbubbles and cell surfaces were measured by analyzing the fluorescent images.

### Fluorescent observation of membrane permeabilization and cytoskeleton disassembly

Propidium Iodide (PI; P4170, Sigma-Aldrich, St. Louis, MO, USA, excitation/emission maxima: 551/670 nm) was chosen as the fluorescence marker to identify sonoporated cells *in-situ*. It was added to the OptiCell™ sample holder at a concentration of 0.25 μg/ml and incubation was performed for 5 min to establish equilibrium before adding the microbubbles. It is well known that PI is membrane impermeant and is only taken up by a cell when its membrane permeability increases. It produces fluorescence by chemical reaction with nucleic^58^ and its intracellular fluorescence would reach a stable plateau once the membrane permeability returns to the homoeostatic level. Hence, it can be used as an indicator for sonoporation that is marked by a transitory increase in the membrane permeability^11,58^.

The protocol used in our experiments involved only fluorescence imaging without the bright-field imaging. The wavelengths of 476 nm and 551 nm were used for GFP and PI fluorescence excitations, respectively. The corresponding detection wavelengths were 510 nm and 670 nm, respectively. Using these parameters, fluorescent frames were acquired every 5 s for a total duration of 4 min.

### Fluorescence images analysis

The time-lapsed fluorescence image frames were then imported into Image-Pro Plus (Media Cybernetics Inc., Bethesda, MD, USA) for quantitative analysis of the sonoporation features. From the acquired images, temporal profiles of the GFP-α-tubulin cytoskeleton arrangement and the membrane permeabilization level were assessed by measuring α-tubulin and PI fluorescence at a frame rate of 12 frames/min over a 3-min observation period. Then, the integrated optical density (IOD) inside the contour of sonoporated cell was estimated using built-in functions in the Image-Pro Plus software. All the measured fluorescence intensities were normalized with respect to corresponding pre-exposure baseline levels. Only cells perforated by a single bubble were selected for analyses.

### A two-dimensional boundary element method (BEM) model of an interacting bubble-cell pair

The initial state of bubble dynamic response is determined by the well-known Rayleigh-Plesset (RP) equation. A free bubble with the spherically symmetrical oscillating mode was adopted for simplicity. The dynamic response of a spherical microbubble sonicated in a flow field can be described with^39^:

3 $${\rho }_{l}({R}_{\text{b}}{\ddot{R}}_{\text{b}}+\frac{3}{2}{\dot{R}}_{\text{b}}^{2})={p}_{\text{g},0}{(\frac{{R}_{\text{b},{\rm{0}}}}{{R}_{\text{b}}})}^{3\kappa }+{p}_{\text{v}}-\frac{2\sigma }{{R}_{\text{b}}}-\frac{4\eta {\dot{R}}_{\text{b}}}{{R}_{\text{b}}}-{p}_{0}-{p}_{\text{ac}}(t),$$

where *R*_b_ was the radius of the bubble as a function of time *t*, with *R*_b,0_ being its equilibrium value, ${\dot{R}}_{{\rm{b}}}=d{R}_{{\rm{b}}}/dt$ and ${\ddot{R}}_{{\rm{b}}}={d}^{2}{R}_{{\rm{b}}}/d{t}^{2}$ being its first- and second-order time derivatives, respectively, *ρ*_1_ was the density of the surrounding liquid. *σ* was the interfacial tension, *κ* is the polytropic exponent of the gas core and *η* was the dilatational viscosity coefficient of the fluid. ${p}_{\text{g},0}={p}_{0}-2\sigma /{R}_{\text{b},{\rm{0}}}+{p}_{\text{v}}$ was the initial pressure inside the bubble, while *p*_0_ and *p*_v_ were the hydrostatic pressure of the surrounding liquid and the saturated vapor pressure of the gas, respectively. *p*_ac_(*t*) was the time-dependent pressure of the exerted ultrasonic field. The initial motion of a given free bubble could be estimated through the RP equation based on its initial radius (*R*_b,0_ = 1.5 µm) and ambient pressure (*p*_0_ = 1.013 × 10^5^ Pa). After the ultrasound began, time variation of symmetrical or asymmetrical bubble oscillation could be obtained through BEM simulations, depending on whether the cell was present at its neighborhood or not.

We treated cells as liquid-filled, compressible spheres from an acoustic perspective and the empirical Tait Equation was selected as its equation of state,

4 $${p}_{{\rm{c}}}=({p}_{0}+B){(\frac{{\rho }_{{\rm{c}}}}{{\rho }_{\text{c},0}})}^{N}-B$$

where *B* and *N* were temperature related constants which could be validated from experiments. *p*_c_ was the pressure inside the cell and *p*_0_ was the reference pressure inside the cell, equal to the ambient pressure outside the microbubble. *ρ*_c_ and *ρ*_c,0_ were densities of the liquid inside the cell at *p*_c_ and *p*_0_, respectively. Considering that volume fraction of water in each cell could be as high as 80%, it was rational to assume properties of the cell resembled those of water, i.e., B = 330.9 MPa, N = 7.15, *ρ*_c,0_ = *ρ*_1_ = 1000 kg/m^3 ^^19^.

*R*_c_ is the time dependent radius of a cell and its equilibrium state value *R*_c,0_ = 10 µm. The cellular surface area was denoted as ${S}_{{\rm{c}}}=4{\boldsymbol{\pi }}{R}_{{\rm{c}}}^{2}$ with its initial value ${S}_{{\bf{c}}{\boldsymbol{,}}0}=4{\boldsymbol{\pi }}{R}_{{\bf{c}}{\boldsymbol{,}}0}^{2}$. Elasticity of the cell membrane was evaluated with a surface tension Tc^39^,

5 $${T}_{\text{c}}=K\frac{{S}_{\text{c}}-{S}_{\text{c}{\boldsymbol{,}}0}}{{S}_{\text{c}{\boldsymbol{,}}0}}$$

which increased linearly with the cellular areal expansion. Areal expansion modulus of the cell, K, was chosen as that of the red blood cell, i.e., K = 0.5 N/m^61^.
